# Long-term clinical outcome of patients with metastatic melanoma and initial stable disease during anti-PD-1 checkpoint inhibitor immunotherapy with pembrolizumab

**DOI:** 10.1038/s41416-025-03048-8

**Published:** 2025-05-26

**Authors:** Inge Mansfield Noringriis, Marco Donia, Kasper Madsen, Henrik Schmidt, Charlotte Aaquist Haslund, Lars Bastholt, Inge Marie Svane, Eva Ellebaek

**Affiliations:** 1 National Center for Cancer Immune Therapy (CCIT-DK), Department of Oncology, Copenhagen University Hospital, Herlev, Denmark; 2 Department of Oncology, Aarhus University Hospital, Aarhus, Denmark; 3 Department of Oncology, Aalborg University Hospital, Aalborg, Denmark; 4 Department of Oncology, Odense University Hospital, Odense, Denmark

**Keywords:** Cancer immunotherapy, Melanoma, Outcomes research, Epidemiology

## Abstract

**Background:**

A substantial number of patients with metastatic melanoma (MM) treated with anti-PD-1 monotherapy have initial stable disease (SD), yet the real-world prognosis of these patients remains unclear.

**Methods:**

In this nationwide cohort study, we analysed real-world outcomes of patients with MM treated with pembrolizumab in Denmark. Focusing on patients with initial SD, we assessed best overall response (BOR), progression-free survival (PFS), and overall survival (OS) and identified predictors of survival in multivariable analyses.

**Results:**

Out of 1048 included patients, 233 (22.2%) had initial SD with a median PFS and OS of 14.7 and 50.1 months. Subsequent partial response (PR) or complete response (CR) was developed by 44 (18.9%) and 52 (22.3%) patients showing significantly improved PFS compared to patients with continued SD (PR: HR 0.52, 95% CI 0.34–0.81, *p* = 0.003; CR: HR 0.15, 95% CI 0.07–0.32, *p* < 0.001) and survival rates comparable to patients with initial PR and CR, respectively. Furthermore, 49 (21.0%) patients showed continued disease control (median follow-up of 82.3 months). For 51.0% of these patients, the last dose of pembrolizumab was administered during SD with a median treatment duration of 12.4 months.

**Conclusions:**

Of patients with initial SD, 40% developed a subsequent objective response with improved long-term prognosis comparable to patients with initial response. More than 20% exhibited continued disease control.

## Introduction

Immune checkpoint inhibitors have dramatically improved the prognosis of patients with metastatic melanoma (MM) by introducing the possibility of durable responses and long-term survival [1–4]. Anti-Programmed Cell Death Protein 1 (PD-1) antibodies achieve their anti-tumour effect through an interruption of the PD-1-mediated inhibition of T-cells in peripheral tissue performed by Programmed Cell Death Ligand 1 (PD-L1) expressing tumour cells [5]. Anti-PD-1 targeting therapy is a major backbone of first-line immunotherapy for MM. Pembrolizumab is one of two anti-PD-1 antibodies approved in this setting [6, 7] with an objective response rate of approximately 40% in clinical trials [1].

Approximately 30% of patients with MM exhibit stable disease (SD) on their first evaluation scan during treatment with first-line pembrolizumab [8]. According to the Response Evaluation Criteria in Solid Tumors (RECIST), SD is defined as a change in tumour size that is neither a partial response (PR) (>30% decrease) nor progressive disease (PD) (>20% increase) [9]. SD is generally associated with poorer survival outcomes compared to PR or complete response (CR) [2, 3, 8, 10]. However, the group of patients with initial SD comprises several prognostically distinct subgroups, including patients with slow progression as well as patients with not (yet) distinguishable responses [11]. In the KEYNOTE-001 and -006 trials, approximately half of the patients with pembrolizumab-induced initial SD eventually did achieve an objective response (OR) [10] with long-term survival rates comparable to patients with continued PR [10].

Real-world data are crucial to validate such prognostic observations from clinical trials in broader patient populations [12]. The long-term outcomes of patients with immune checkpoint inhibitor-induced initial SD remain to be elucidated in a real-world setting. Consequently, SD represents a prognostic grey area where patient outcomes are not easily predicted. Identification of prognostic markers indicative of either continued disease control (cDC) or subsequent progression is needed to further improve the treatment and guidance of patients with MM and initial SD. Here, we investigate the long-term clinical outcomes of patients with MM and initial pembrolizumab-induced SD in a nationwide, real-world setting.

## Materials and methods

### Study design and population

The Danish Metastatic Melanoma Database (DAMMED) is a nationwide registry including disease, treatment, and outcome data on all patients with melanoma considered for medical therapy in Denmark [13]. Written informed consent is obtained from all participants. Data are collected through the evaluation of hospital files by trained medical students and are selectively controlled by responsible clinicians. Response status registered in DAMMED is derived from RECIST-based evaluations of Computed Tomography (CT) or Fluorodeoxyglucose Positron Emission Tomography (FDG-PET)/CT scans combined with clinical assessments by the physicians.

We selected patients diagnosed with unresectable stage III or IV cutaneous melanoma or melanoma of unknown primary, treated with pembrolizumab monotherapy in any line between July 2014 and April 2022. In this period, pembrolizumab was the standard anti-PD-1 antibody used as monotherapy for unresectable MM in Denmark, as recommended by the Danish Medical Council. Patients were followed until the lock of the database on April 12th, 2024 (data cut-off). Initial response was defined as the response at the first evaluation scan between 6 and 18 weeks after initiation of therapy. Continued disease control (cDC) was defined as being alive and progression-free at the time of data cut-off with a minimum progression-free survival (PFS) of 24 months. Outcomes were best overall response (BOR), cDC, PFS, and overall survival (OS).

### Statistical analysis

We used R version 4.3.2 for all statistical analyses. The limit for statistical significance was defined as a two-sided *p*-value of <0.05. Follow-up time was calculated by the reverse Kaplan–Meier method.

Clinical baseline characteristics of patients with initial SD were compared to patients with initial OR or initial PD using Pearson’s Chi-squared Test for categorical variables. Age distribution was compared using the Kruskal–Wallis Rank Sum Test, following the identification of a skewed distribution as assessed by histograms and Q-Q plots. Post-hoc comparisons using Fisher’s Exact Test were made for the significantly differing baseline characteristics and adjusted for multiple tests by Holm’s method.

Survival curves with OS and PFS were calculated using the Kaplan–Meier method with landmark analyses to avoid immortal time bias. Patients who did not die or progress were censored at the last day known to be alive or progression-free. Patients were compared based on their response status at the specific landmark time point, while patients experiencing an event (progression or death) before the landmark were excluded from the analysis. We defined two landmark models: In the first, survival outcomes were stratified by the response status at 18 weeks after initiation of therapy (the 4-month landmark), representing the end of the defined interval for the first evaluation scan and thereby the initial response. In the second, survival outcomes for the patients with initial SD were stratified by the response status at 12 months after initiation of therapy (the 12-month landmark). The 12-month landmark model also included comparisons of the outcomes of patients with initial SD and subsequent OR to the patients with initial and continued OR. Hazard ratios (HR) were calculated using Cox proportional hazards regression models.

We assessed the PFS benefit of achieving OR after initial SD through a multivariable Cox regression analysis with response included as a time-varying effect using SD as reference. It was performed as a complete case analysis, excluding variables with more than 25% missing data, and stratified by line of therapy.

We analysed the associations between potential prognostic factors and PFS and OS through univariable and multivariable Cox regression analyses including the following clinically relevant variables: sex, age, any *BRAF* mutation or wildtype, PD-L1 status < or ≥1%, pembrolizumab given in 1st or ≥2nd line of therapy, American Joint Committee on Cancer (AJCC) stage M1a, M1b, M1c or M1d, lactate dehydrogenase (LDH) level ≤ or > the upper limit of normal, and Eastern Cooperative Oncology Group Performance Status (PS) 0 or ≥1. We used multiple imputations for missing data. Furthermore, we assessed the association between initial metabolic response and PFS in a multivariable analysis of patients from the Eastern part of Denmark evaluated by FDG-PET/CT. The initial FDG-PET/CT scan reports were reviewed by the investigator (IMN). Based on the described changes in metabolic activity, we categorised the patients as having either decreasing or non-decreasing metabolism. Lesions, primarily lymph nodes, suspicious of inflammation or sarcoidosis, were not considered indicators of progression. The model included the same variables as mentioned above. For all Cox regression analyses, compliance with the proportional hazards assumption was evaluated with Schoenfeld residuals and Schoenfeld’s global test.

## Results

### Population and baseline characteristics

**Fig. 1:**
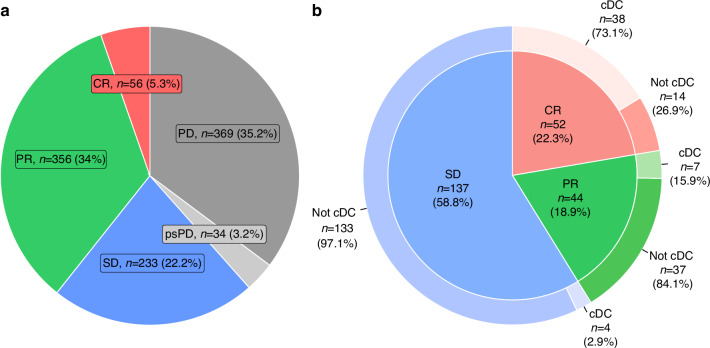
Response status following pembrolizumab monotherapy. **a** Distribution of the total population (*n* = 1048) by response status at the first evaluation scan. **b** Distribution of patients with initial SD (*n* = 233) by the best overall response and achievement of continued disease control, defined as being alive and progression-free at the time of data cut-off with a PFS > 24 months. CR complete response, PR partial response, SD stable disease, psPD pseudoprogression, PD progressive disease, cDC continued disease control.

A total of 1048 patients with MM treated with pembrolizumab between July 2014 and April 2022 were included (Supplementary Fig. 1). The median follow-up time was 72.1 months (IQR 50.5–93.1 months). The first evaluation scan was performed after a median of 11 weeks (IQR 10.0–11.9 weeks), and here 233 patients (22.2%) had SD (Fig. 1a). Among the 154 patients who underwent their first evaluation scan within 9 weeks of treatment initiation, the majority (*n* = 128, 83.1%) were evaluated due to clinical signs of early PD. Of the remaining patients, 13 (8.4%) had SD, 12 (7.8%) had PR, and 1 (0.6%) had CR. Most patients with initial SD were *BRAF* wildtype (59.3%) and were treated in the first line of therapy (64.8%) (Table 1). Compared to patients with initial OR, significantly more patients with initial SD were PD-L1 negative (48.9% versus 34.8%, *p* = 0.045) and treated in second or later lines of therapy (35.2% versus 17.2%, *p* < 0.001). The AJCC stages differed significantly between patients with initial SD and initial OR, with more having stage M1a, M1c, and M1d (30.5% versus 25.0%, 41.2% versus 39.8%, and 13.3% versus 10.2%), and fewer having stage M1b (15.0% versus 25.0%) (*p* = 0.017). Furthermore, PD-L1 expression, AJCC stage, LDH level, and PS differed significantly between patients with initial SD and those with initial PD (Table 1 and Supplementary Table 1).

**Table 1:** Baseline characteristics.

		Response at first evaluation			
		OR	SD	PD	*P*-value
**Number of patients**		412	233	369	
**Sex**	Female	159 (38.6%)	101 (43.3%)	142 (38.5%)	0.420
	Male	253 (61.4%)	132 (56.7%)	227 (61.5%)	
**Age**	Mean	69.3 (12.1)	67.2 (12.9)	67.9 (13.4)	0.111
**BRAF status**	Wildtype	242 (59.3%*)	134 (59.3%*)	213 (57.9%*)	0.907
	Mutated	166 (40.7%*)	92 (40.7%*)	155 (42.1%*)	
	Not tested	4	7	1	
**PD-L1 status**	<1%	77 (34.8%*)	45 (48.9%*)	104 (63.0%*)	<0.001
	≥1%	144 (65.2%*)	47 (51.1%*)	61 (37.0%*)	
	Unknown	191	141	204	
**Line of therapy**	1st	341 (82.8%)	151 (64.8%)	246 (66.7%)	<0.001
	≥2nd	71 (17.2%)	82 (35.2%)	123 (33.3%)	
**AJCC stage**	M1a	103 (25.0%)	71 (30.5%)	68 (18.4%)	<0.001
	M1b	103 (25.0%)	35 (15.0%)	41 (11.1%)	
	M1c	164 (39.8%)	96 (41.2%)	164 (44.4%)	
	M1d	42 (10.2%)	31 (13.3%)	96 (26.0%)	
**LDH**	≤ULN	234 (59.2%*)	130 (57.0%*)	144 (40.7%*)	<0.001
	>ULN	161 (40.8%*)	98 (43.0%*)	210 (59.3%*)	
	Unknown	17	5	15	
**ECOG PS**	0	250 (60.7%)	135 (57.9%)	160 (43.4%)	<0.001
	≥1	162 (39.3%)	98 (42.1%)	209 (56.6%)	

Values are *n* (%) for categorical variables and *mean* (standard deviations) for quantitative variables. *P*-values are calculated by Pearson’s Chi-squared Test for categorical variables and Kruskal–Wallis Rank Sum Test for quantitative variables.

*OR* objective response, *SD* stable disease, *PD* progressive disease, *PD-L1* programmed cell death ligand 1, *AJCC stage* American Joint Committee on Cancer stage, *LDH* lactate dehydrogenase, *ULN* upper limit of normal, *ECOG PS* Eastern Cooperative Oncology Group performance status.

*Percentages excluding patients with unknown results.

### Outcome for patients with initial SD

Of the 233 patients with initial SD, 96 patients (41.2%) obtained a BOR of either PR (44, 18.9%) or CR (52, 22.3%) (Fig. 1b). The median time from initiation of therapy to PR was 5.6 months and to CR 13.7 months (Supplementary Fig. 2). The remaining 137 patients (58.8%) had BOR SD. Of patients with initial SD, 49 (21.0%) had cDC after a median follow-up of 82.3 months (IQR 56.6–96.3 months). cDC was obtained by 38 patients (73.1%) with BOR CR, 7 patients (15.9%) with BOR PR, and 4 patients (2.9%) with BOR SD (Fig. 1b). Out of the 58 patients with SD at the 12-month landmark, cDC was obtained by 18 (31.0%) (data not shown). For the patients achieving cDC, the median duration of pembrolizumab treatment was 12.4 months (IQR 8.1–16.8 months). Only two patients achieving cDC were treated beyond 24 months (one with BOR CR and one with BOR SD). For 25 patients (51.0%) with cDC, the last dose of pembrolizumab was administered during SD; 19 with BOR CR, 2 with BOR PR, and 4 with BOR SD.

### SD at the 4-month landmark

**Fig. 2:**
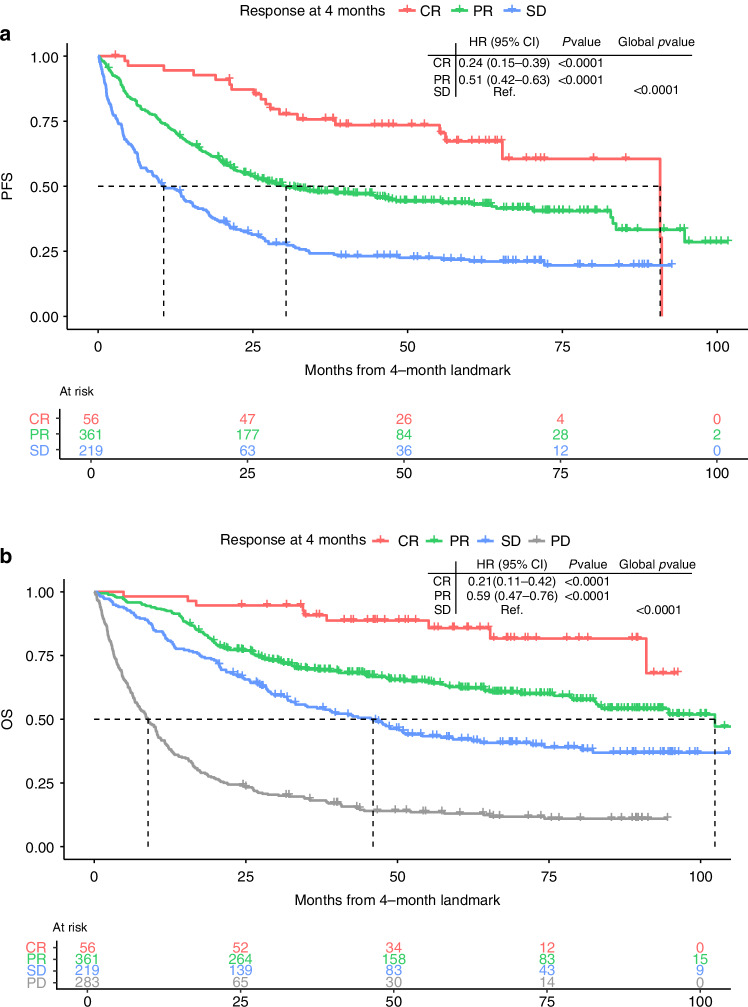
Kaplan–Meier estimates of survival for the total population stratified by response status at the 4-month landmark. **a** PFS. **b** OS. PFS progression-free survival, OS overall survival, CR complete response, PR partial response, SD stable disease, PD progressive disease, HR hazard ratio, 95% CI 95% confidence interval, Global *p*-value likelihood ratio test.

The 4-month landmark represents the end of the defined interval of the first evaluation scan. Patients with SD at the 4-month landmark had worse survival rates than patients who at the 4-month landmark showed PR (PFS: HR 0.51, 95% confidence interval (95% CI) 0.42–0.63, *p* < 0.0001; OS: HR 0.59, 95% CI 0.47–0.76, *p* < 0.0001) or CR (PFS: HR 0.24, 95% CI 0.15–0.39, *p* < 0.0001; OS: 0.21, 95% CI 0.11–0.42, *p* < 0.0001) (Fig. 2a, b). For patients with SD at the 4-month landmark, the median PFS (mPFS) from initiation of therapy was 14.7 months, and the median OS (mOS) was 50.1 months. For patients with initial PR, the mPFS was 34.5 months and the mOS 106.5 months, and for patients with initial CR, the mPFS was 94.9 months while the mOS was not reached. Furthermore, for patients with SD at the 4-month landmark, the 5-year PFS and OS rates from initiation of therapy were 21.8% (95% CI 16.8–28.4) and 43.4% (95% CI 37.0–50.8) (The corresponding 1-, 3-, and 5-year PFS and OS stratified by response status at the 4-month landmark are presented in Supplementary Table 2A+2B).

### Initial SD and response at the 12-month landmark

**Fig. 3:**
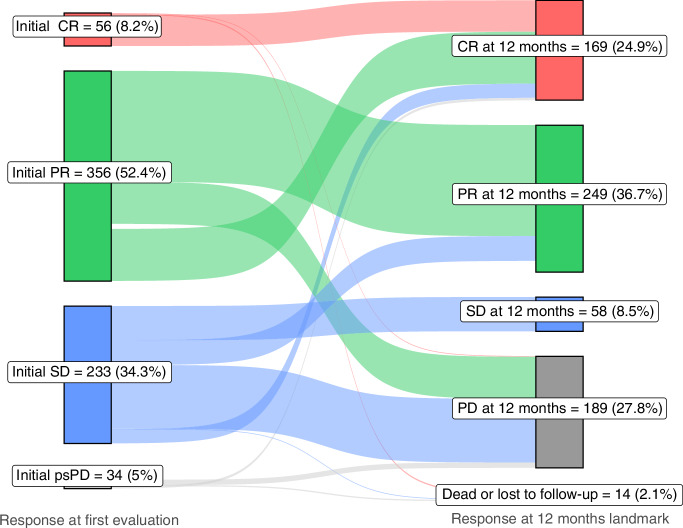
Sankey diagram of changes in response status between the first evaluation scan and the 12-month landmark for the total population. CR complete response, PR partial response, SD stable disease, psPD pseudoprogression, PD progressive disease.

Of the 233 patients with initial SD, 175 (75.1%) changed response status before the 12-month landmark (Fig. 3). By the 12-month landmark, 66 (28.3%) patients with initial SD had achieved an OR - 24 patients (10.3%) had CR and 42 (18.0%) had PR - while 109 (46.8%) patients had progressed or died, and one patient was lost to follow-up.

**Fig. 4:**
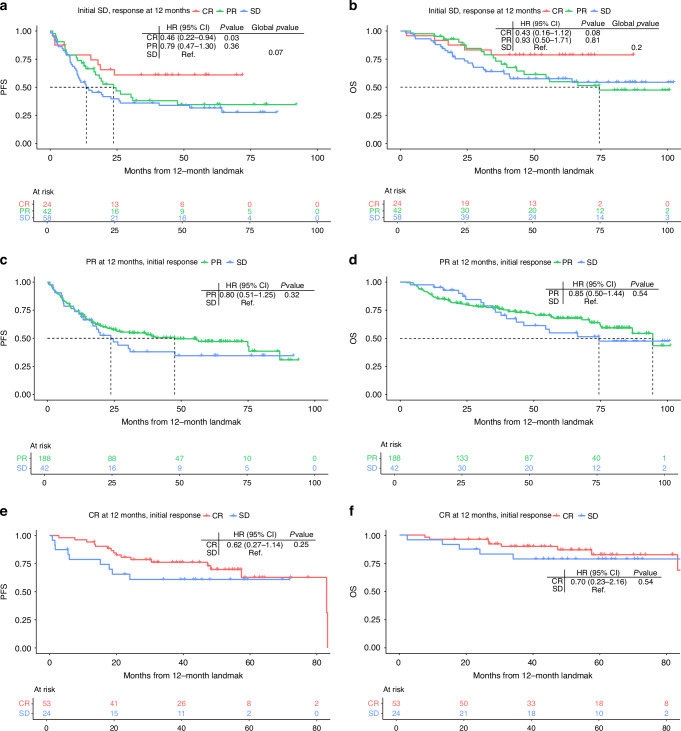
Kaplan–Meier estimates of survival. **a** PFS of patients with initial SD stratified by response status at the 12-month landmark. **b** OS of patients with initial SD stratified by response status at the 12-month landmark. **c** PFS for patients with PR at the 12-months landmark stratified by initial response status. **d** OS for patients with PR at the 12-months landmark stratified by initial response status. **e** PFS for patients with CR at the 12-months landmark stratified by initial response status. **f** OS for patients with CR at the 12-months landmark stratified by initial response status. PFS progression-free survival, OS overall survival, CR complete response, PR partial response, SD stable disease, HR hazard ratio, 95% CI 95% confidence interval, Global *p*-value likelihood ratio test.

Patients with initial SD achieving subsequent CR before the 12-month landmark had significantly superior PFS compared to patients with continued SD (HR 0.46, 95% CI 0.22–0.94, *p* = 0.03) and a trend towards superior OS (HR 0.43, 95% CI 0.16–1.12, *p* = 0.08) (Fig. 4a, b). No significant difference was seen for patients achieving subsequent PR (PFS: HR 0.79, 95% CI 0.47–1.30, *p* = 0.36; OS: HR 0.93, 95% CI 0.50–1.71, *p* = 0.81). For patients achieving CR, neither mPFS nor mOS were reached, whereas the 5-year PFS and OS rates (from initiation of therapy) were 60.9% (95% CI 43.9–84.6) and 78.7% (95% CI 63.8–97.2), respectively (Supplementary Table 3A+3B). In comparison, for patients with continued SD at the 12-month landmark, the mPFS was 25.5 months (95% CI 22.4–52.8), the mOS was not reached, the 5-year PFS rate was 33.8% (95% CI 23.3–49.1) and the 5-year OS rate was 57.6% (95% CI 45.7–72.6). By the 12-month landmark, 108 patients with initial SD had progressed, of whom 77 (71.3%) were still alive. These patients who progressed after an initial SD had a mOS of 26.6 months (95% CI 22.4–33.4) and a 5-year OS rate of 23.2% (95% CI 15.1–35.6) (Supplementary Table 3A+3B).

Patients with initial OR and continued response at the 12-month landmark had comparable - trending towards slightly better - survival rates compared to patients with initial SD achieving an OR before the 12-month landmark (PR: PFS: HR 0.80, 95% CI 0.51–1.25, *p* = 0.32; OS: HR 0.85, 95% CI 0.50–1.44, *p* = 0.54) (CR: PFS: HR 0.62, 95% CI 0.27–1.41, *p* = 0.25; OS: HR 0.70, 95% CI 0.23–2.16, *p* = 0.54) (Fig. 4c–f).

**Fig. 5:**
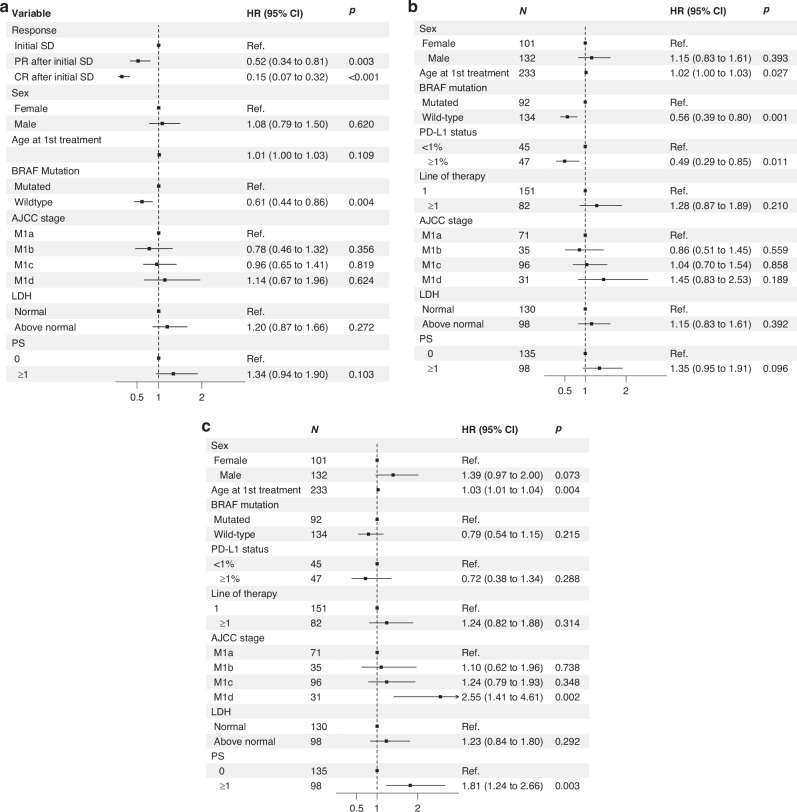
Multivariable Cox regression analysis for patients with initial stable disease. **a** Time to PFS, including response as a time-varying effect using SD as reference. **b** Time to PFS, including PD-L1, with multiple imputations used for missing data. **c** Time to OS, including PD-L1, with multiple imputations used for missing data. PD-L1 programmed cell death ligand 1, AJCC stage American Joint Committee on Cancer stage, LDH lactate dehydrogenase, PS Eastern Cooperative Oncology Group performance status.

In the multivariable analysis with time-varying covariates, achieving PR after initial SD reduced the hazard of progression by 48% (HR 0.52, 95% CI 0.34–0.81, *p* = 0.003), while achieving CR reduced the hazard by 85% (HR 0.15, 95% CI 0.07–0.32, *p* < 0.001) (Fig. 5a).

### Prognostic markers of patients with initial SD

In an univariable analysis, *BRAF* status, PD-L1 expression, and PS at baseline significantly correlated to PFS, while age, AJCC stage M1c and M1d, baseline LDH level, and PS significantly correlated to OS (Supplementary Table 4). The multivariable analysis of PFS showed *BRAF* wildtype status (HR 0.56, 95% CI 0.39–0.80, *p* = 0.001) and PD-L1 expression ≥1% (HR 0.49, 95% CI 0.29–0.85, *p* = 0.011) as significant positive predictive factors of PFS (Fig. 5b). Negative predictive factors of OS in the multivariable analysis were AJCC stage M1d (HR 2.55, 95% CI 1.41–4.61, *p* = 0.002) and PS ≥ 1 (HR 1.81, 1.24–2.66, *p* = 0.003) (Fig. 5c).

Of the 233 patients with initial SD, 95 (40.8%) were included in the multivariable analysis of patients evaluated by FDG-PET/CT scans. Of these, 19 patients (20%) showed a decrease in metabolism at the initial evaluation scan which was numerically associated with prolonged PFS (HR 0.51, 95% CI 0.24–1.08, *p* = 0.077) (Supplementary Fig. 3). Of the 19 patients with initial SD and concomitant decreasing metabolism, 11 (57.9%) subsequently obtained OR compared to 23 (30.3%) of the 76 patients with initial SD and non-decreasing metabolism.

## Discussion

In this nationwide, real-world cohort of patients with MM, we observed that a noticeable proportion of patients treated with pembrolizumab had initial SD and that 40% of these patients later obtained an OR. Compared to patients with continued SD, these patients with subsequent OR demonstrated improved survival rates, which were comparable to the patients with initial OR.

In our cohort, 22.2% of patients showed SD on their first evaluation scan, which is comparable to the 24%–28% found in other real-world cohorts of patients with MM treated with anti-PD-1 monotherapy [4, 8]. We observed that patients exhibiting SD by the 4-month landmark had significantly worse PFS and OS rates compared to patients with PR or CR, also aligning with previous findings [2, 3, 8, 10]. The group of patients with initial SD was dynamic, with three out of four patients demonstrating a change in response status after 12 months −22.3% eventually achieving BOR CR and 18.9% achieving BOR PR. In comparison, data from the KEYNOTE-001 and -006 trials found that only 6.5% of patients with initial SD achieved BOR CR, while 40.2% achieved PR [10]. These discrepancies may be attributed to a longer follow-up in our study, combined with stricter adherence to the RECIST criteria in clinical trials. Notably, the fraction of patients with initial SD achieving subsequent OR was comparable with 41.2% in our cohort and 46.7% in the KEYNOTE trials.

Developing subsequent OR improved the prognosis of patients with initial SD significantly. In the multivariable analysis with time-varying covariates, the change of response from SD to PR halved the risk of progression, while the achievement of CR decreased the risk by 85%. Especially, patients achieving subsequent CR appear to obtain superior survival outcomes with significantly improved PFS and a trend towards improved OS compared to patients with continued SD. The superior prognosis of these patients was further supported by the observation that 38 out of 49 (77.6%) patients with cDC had BOR CR. Importantly, we found that the survival rates of patients with initial SD and subsequent OR were comparable to the survival rates of patients with initial OR. Among patients who achieved cDC, the median duration of treatment was one year. This supports the potential for long-term survival following immune checkpoint inhibitor discontinuation, consistent with previous findings [14]. While prior studies have associated the achievement of CR during treatment with improved prognosis compared to patients with PR or SD at treatment discontinuation [14], we observed that half of the patients achieving cDC discontinued pembrolizumab while in SD. Notably, most of these patients subsequently developed CR after pembrolizumab discontinuation, suggesting that the timing of BOR CR may not be as prognostically important as previously thought.

The identification of predictive factors is needed to improve the treatment of patients with initial SD. In multivariable analysis, we found that the presence of a *BRAF* mutation and PD-L1 expression <1% were significantly associated with a shorter PFS compared to patients with *BRAF* wildtype and PD-L1 expression ≥1%. Our results support previous findings that *BRAF-*mutated melanomas display a more aggressive phenotype than *BRAF* wild-type melanomas [15–18]. This association was, however, not seen in the multivariable analysis of OS, presumably due to the availability of targeted therapy as an additional treatment option for this group of patients. The value of PD-L1 tumour expression as a biomarker has been intensively discussed, and several studies have observed a correlation between the level of PD-L1 expression and prognosis [19–22]. However, there are limitations to the utility of PD-L1 as a biomarker, including pronounced intra-tumoral, intra-patient, and temporal heterogeneity of PD-L1 expression [21, 23]. In the multivariable analysis of OS, we found that baseline characteristics of AJCC stage M1d and PS ≥ 1 were significantly associated with shorter OS, in line with previous findings [24]. In a sub-analysis, we found that initial SD with a concomitant decrease in FDG-metabolism was numerically associated with prolonged PFS. Though not significant, this finding does add to a growing body of evidence that the metabolic response assessed by PET/CT scans at various time points is prognostic of response and long-term survival [25–28]. Thus, our results show that, in patients with initial SD, having a *BRAF* wildtype status, a PD-L1 expression ≥1%, and initial decreasing metabolism indicate prolonged response to pembrolizumab.

Our study has some limitations: the response status registered in DAMMED is retrospectively transcribed from patient files and based on the treating oncologists’ overall evaluation of scan reports provided by radiologists. These evaluations are generally based on the RECIST principles but are also, to some extent, influenced by clinical assessments. Consequently, adherence to the RECIST criteria is generally not as strict as can be expected in randomised clinical trials. Furthermore, missing data on PD-L1 expression poses a limitation in the multivariable analyses, and in the subgroup analysis of patients evaluated by PET/CT scans, only patients from the Eastern part of Denmark were included, resulting in a smaller patient cohort.

## Conclusion

In this real-world study including 1048 patients with MM treated with pembrolizumab monotherapy and a median follow-up of 72.1 months, we found that among 233 (22%) patients with initial SD, 40% achieved a subsequent OR resulting in an improved prognosis compared to patients with BOR SD and survival outcomes comparable to patients with an initial OR. Continued disease control with PFS of more than 24 months was common and achieved by more than 20% of patients with initial SD. Importantly, half of the patients achieving continued disease control discontinued pembrolizumab while in SD. Key good prognostic factors for prolonged PFS included having a non-*BRAF*-mutated tumour, PD-L1 expression ≥1%, and initial metabolic response. Further insight into the immunological characteristics of patients with continued disease control or delayed response during treatment with immune checkpoint inhibitors is highly wanted.

## Supplementary information

Supplementary material

## Data Availability

The data that support the findings of this study can be made available via application to the Danish Metastatic Melanoma Database steering committee. Further details are available from the corresponding authors upon request.
