# Supplementary material for: Long-term clinical outcome of patients with metastatic melanoma and initial stable disease during anti-PD-1 checkpoint inhibitor immunotherapy with pembrolizumab
Source: Br J Cancer. 2025 May 26;133(3):337–45. doi: 10.1038/s41416-025-03048-8 (PMC12322104; doi:10.1038/s41416-025-03048-8)
Supplement: Supplementary file 1 — Supplementary material [file 41416_2025_3048_MOESM1_ESM.docx]

**Supplementary Material**

**Title**

Long-term clinical outcome of patients with metastatic melanoma and initial stable disease during anti-PD-1 checkpoint inhibitor immunotherapy with pembrolizumab

**Authors**

Inge Mansfield Noringriis, Marco Donia, Kasper Madsen, Henrik Schmidt, Charlotte Aaquist Haslund, Lars Bastholt, Inge Marie Svane, Eva Ellebaek

**Content**

Page 2: **Figure S1:** Flow chart of the selection of patients for analysis.

Page 3: **Table S1:** Post-hoc comparisons of the significantly differing baseline variables.

Page 4: **Figure S2:** Violin plot of time to PR and CR from initiation of therapy for patients with initial stable disease.

Page 5: **Table S2:** Survival outcomes for the total population stratified by response status at the 4-month landmark.

Page 6: **Table S3:** Survival outcomes for the total population stratified by response status at the 12-month landmark.

Page 7: **Table S4:** Univariable Cox Regression analysis of PFS and OS for patients with initial stable disease.

Page 8: **Figure S3:** Subgroup multivariable Cox Regression analysis of PFS for patients with initial stable disease evaluated by PET-CT scan in the Eastern part of Denmark.

**Supplementary Figure S1:**

**
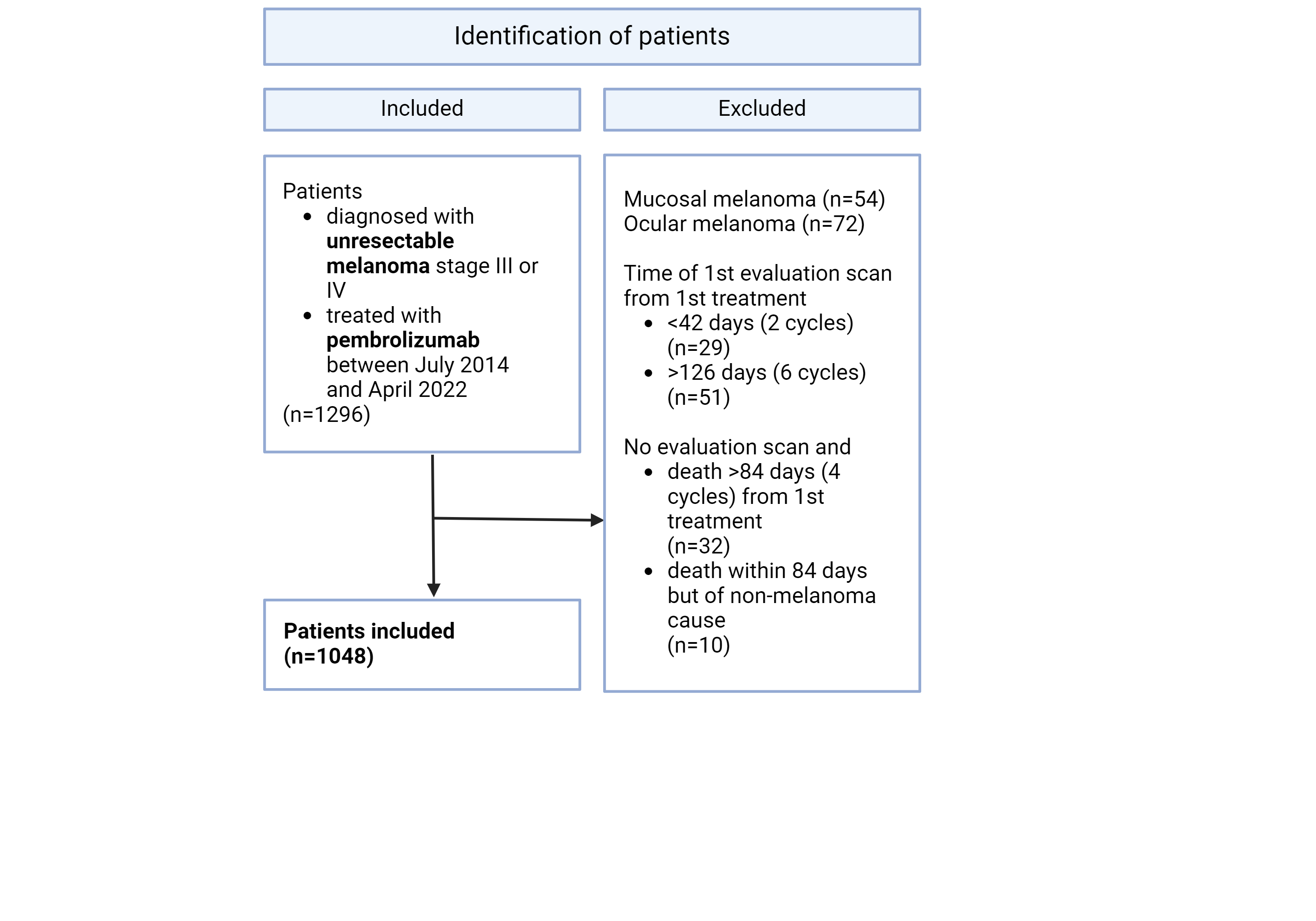
**

**Figure S1:** Flow chart of the selection of patients for analysis.

**Supplementary Table 1**

|  |  | **OR vs. SD** | | **OR vs. PD** | | **SD vs. PD** | |
| --- | --- | --- | --- | --- | --- | --- | --- |
|  |  | **Unadjusted** | **Adjusted** | **Unadjusted** | **Adjusted** | **Unadjusted** | **Adjusted** |
| **PD-L1 status** | <1% | 0.022 | 0.045 | <0.001 | <0.001 | 0.035 | 0.045 |
|  | ≥1% |  |  |  |  |  |  |
| **Line of therapy** | 1st | <0.001 | <0.001 | <0.001 | <0.001 | 0.659 | 0.659 |
|  | ≥2nd |  |  |  |  |  |  |
| **AJCC stage** | M1a | 0.017 | 0.017 | <0.001 | <0.001 | <0.001 | <0.001 |
|  | M1b |  |  |  |  |  |  |
|  | M1c |  |  |  |  |  |  |
|  | M1d |  |  |  |  |  |  |
| **LDH** | ≤ULN | 0.613 | 0.613 | <0.001 | <0.001 | <0.001 | <0.001 |
|  | >ULN |  |  |  |  |  |  |
| **ECOG PS** | 0 | 0.505 | 0.505 | <0.001 | <0.001 | 0.001 | 0.001 |
|  | ≥1 |  |  |  |  |  |  |

**Table S1:** Post-hoc comparisons of the significantly differing baseline variables with P-values calculated by Fisher’s Exact Test. Holm adjustment for multiple tests within each baseline variable.

OR: objective response; SD: stable disease; PD: progressive disease; PD-L1, Programmed Cell Death Ligand 1; AJCC stage, American Joint Committee on Cancer stage; LDH, lactate dehydrogenase; ULN, upper limit of normal; ECOG PS, Eastern Cooperative Oncology Group performance status.

**Supplementary Figure S2:**

**
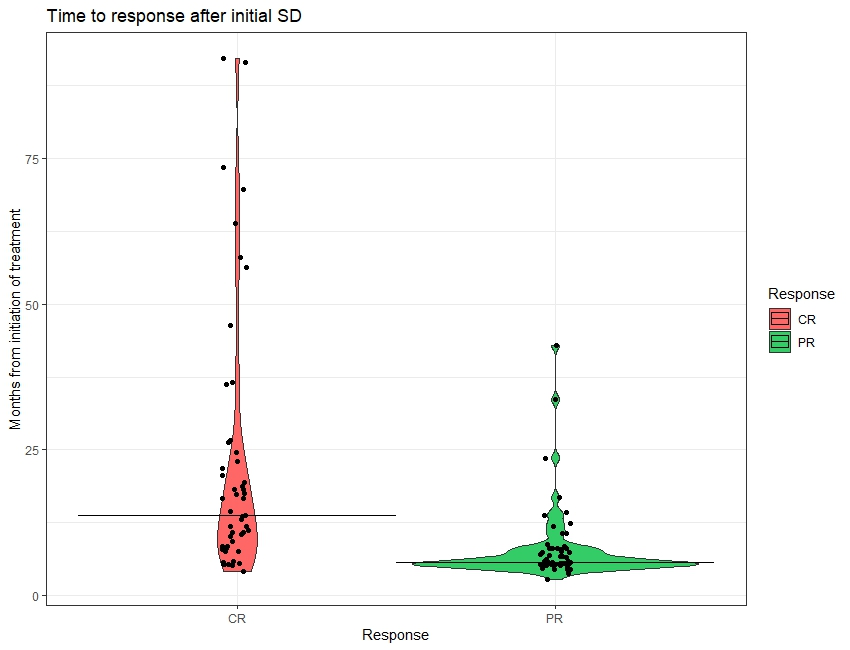
**

**Figure S2:** Violin plot of time to PR and CR from initiation of therapy for patients with initial stable disease. The horizontal lines mark the median time to response.

CR, complete response; PR, partial response.

**Supplementary Table S2A:**

|  | **PFS (95% CI)** | | | |
| --- | --- | --- | --- | --- |
| **Response at 4-month landmark (n)** | **Median** | **1-year** | **3-year** | **5-year** |
| CR (56) | 94.9  (69.4-NR) | 96.4  (91.5-100) | 77.7  (67.3-89.7) | 70.4  (58.6-84.6) |
| PR (361) | 34.5  (27.6-59.6) | 78.9  (74.8-83.2) | 49.2  (44.2-54.8) | 43.6  (38.5-49.5) |
| SD (219) | 14.7  (12.0-19.4) | 56.6  (50.4-63.6) | 26.8  (21.5-33.5) | 21.8  (16.8-28.4) |

**Supplementary Table S2B:**

|  | **OS (95% CI)** | | | |
| --- | --- | --- | --- | --- |
| **Response at 4-month landmark (n)** | **Median** | **1-year** | **3-year** | **5-year** |
| CR (56) | NR | 98.2  (94.8-100) | 94.6  (88.9-100) | 85.8  (76.3-96.4) |
| PR (361) | 106.5  (86.9-NR) | 95.3  (93.1-97.5) | 71.8  (67.3-76.7) | 64.1  (59.0-69.5) |
| SD (219) | 50.1  (37.4-63.3) | 89.5  (85.5-93.7) | 58.3  (52.1-65.3) | 43.4  (37.0-50.8) |
| PD (283) | 13.0  (11.3-14.5) | 53.4  (47.9-59.5) | 20.0  (15.9-25.3) | 13.5  (10.0-18.4) |

**Table S2:** Survival outcomes for the total population stratified by response status at the 4-month landmark.

A: PFS from initiation of therapy

B: OS from initiation of therapy

PFS, progression-free survival; OS, overall survival; 95%CI, 95% confidence interval; NR, not reached; CR, complete response; PR, partial response; SD, stable disease; PD, progressive disease.

**Supplementary Table S3A:**

|  |  | **PFS (95% CI)** | | |
| --- | --- | --- | --- | --- |
| **Response at 12-month landmark** | **Response at 1^st^ evaluation** | **Median** | **3-year** | **5-year** |
| CR | CR (53) | 94.9  (69.4-NR) | 80.6  (70.5-92.2) | 73.1  (61.3-87.1) |
|  | SD (24) | NR | 65.6  (48.9-88.1) | 60.9  (43.9-84.6) |
| PR | PR (188) | 59.6  (37.7-NR) | 58.1  (51.3-65.8) | 49.6  (42.4-58.0) |
|  | SD (42) | 35.7  (28.9-NR) | 49.7  (36.1-68.3) | 34.5  (21.9-54.3) |
| SD | SD (58) | 25.5  (22.4-52.8) | 39.7  (28.8-54.9) | 33.8  (23.3-49.1) |

**Supplementary Table S3B:**

|  |  | **OS (95% CI)** | | |
| --- | --- | --- | --- | --- |
| **Response at 12-month landmark** | **Response at 1^st^ evaluation** | **Median** | **3-year** | **5-year** |
| CR | CR (53) | NR | 96.2  (91.2-100) | 86.8  (77.3-97.5) |
|  | SD (24) | NR | 87.5  (75.2-100) | 78.7  (63.8-97.2) |
| PR | PR (188) | 106.5  (98.9-NR) | 80.5  (74.9-86.4) | 72.2  (65.7-79.4) |
|  | SD (42) | 86.4  (58.3-NR) | 87.3  (77.4-98.4) | 61.3  (47.2-79.7) |
| SD | SD (58) | NR | 73.4  (62.6-85.9) | 57.6  (45.7-72.6) |
| PD | SD (77) | 26.6  (22.4-33.4) | 37.3  (27.8-49.9) | 23.2  (15.1-35.6) |

**Table S3:** Survival outcomes for the total population stratified by response status at the 12-month landmark.

A: PFS from initiation of therapy

B: OS from initiation of therapy

PFS, progression-free survival; OS, overall survival; 95%CI, 95% confidence interval; NR, not reached; CR, complete response; PR, partial response; SD, stable disease; PD: progressive disease.

**Supplementary Table 4**

|  |  |  | **PFS** | | **OS** | |
| --- | --- | --- | --- | --- | --- | --- |
|  |  | **N** | **HR (95%CI)** | **P-value** | **HR (95%CI)** | **P-value** |
| **Sex** | **Female** | 101 | - | 0.722 | - | 0.537 |
|  | **Male** | 132 | 1.06  (0.78-1.42) |  | 1.11  (0.79-1.56) |  |
| **Age at 1^st^ treatment** |  | 233 | 1.01  (1.00-1.02) | 0.132 | 1.02  (1.01-1.04) | 0.001 |
| **BRAF mutation** | **Mutated** | 92 | - | 0.040 | - | 1.000 |
|  | **Wild-type** | 134 | 0.73  (0.54-0.99) |  | 1.00  (0.71-1.41) |  |
| **PD-L1 status** | **<1%** | 45 | - | 0.030 | - | 0.414 |
|  | **≥1%** | 47 | 0.59  (0.37-0.95) |  | 0.80  (0.47-1.37) |  |
| **Line of therapy** | **1** | 151 | - | 0.280 | - | 0.291 |
|  | **≥2** | 82 | 1.18  (0.87-1.60) |  | 1.20  (0.85-1.70) |  |
| **AJCC stage** | **M1a** | 71 | - |  | - |  |
|  | **M1b** | 35 | 0.91  (0.57-1.45) | 0.684 | 1.31  (0.76-2.28) | 0.327 |
|  | **M1c** | 96 | 1.16  (0.82-1.65) | 0.394 | 1.54  (1.01-2.35) | 0.046 |
|  | **M1d** | 31 | 1.27  (0.79-2.04) | 0.330 | 2.46  (1.44-4.21) | 0.001 |
| **LDH** | **Normal** | 130 | - | 0.161 | - | 0.019 |
|  | **Above normal** | 98 | 1.24  (0.92-1.67) |  | 1.50  (1.07-2.11) |  |
| **PS** | **0** | 135 | - | 0.011 | - | <0.001 |
|  | **≥1** | 98 | 1.47  (1.09-1.98) |  | 2.29  (1.63-3.22) |  |

**Table S4:** Univariable Cox Regression analysis of PFS and OS for patients with initial stable disease.

PFS, progression-free survival; OS, overall survival; HR: hazard ratio; 95%CI, 95% confidence interval; PD-L1, Programmed Cell Death Ligand 1; AJCC stage, American Joint Committee on Cancer stage; LDH, lactate dehydrogenase; PS, Eastern Cooperative Oncology Group performance status.

**Supplementary Figure S3:**


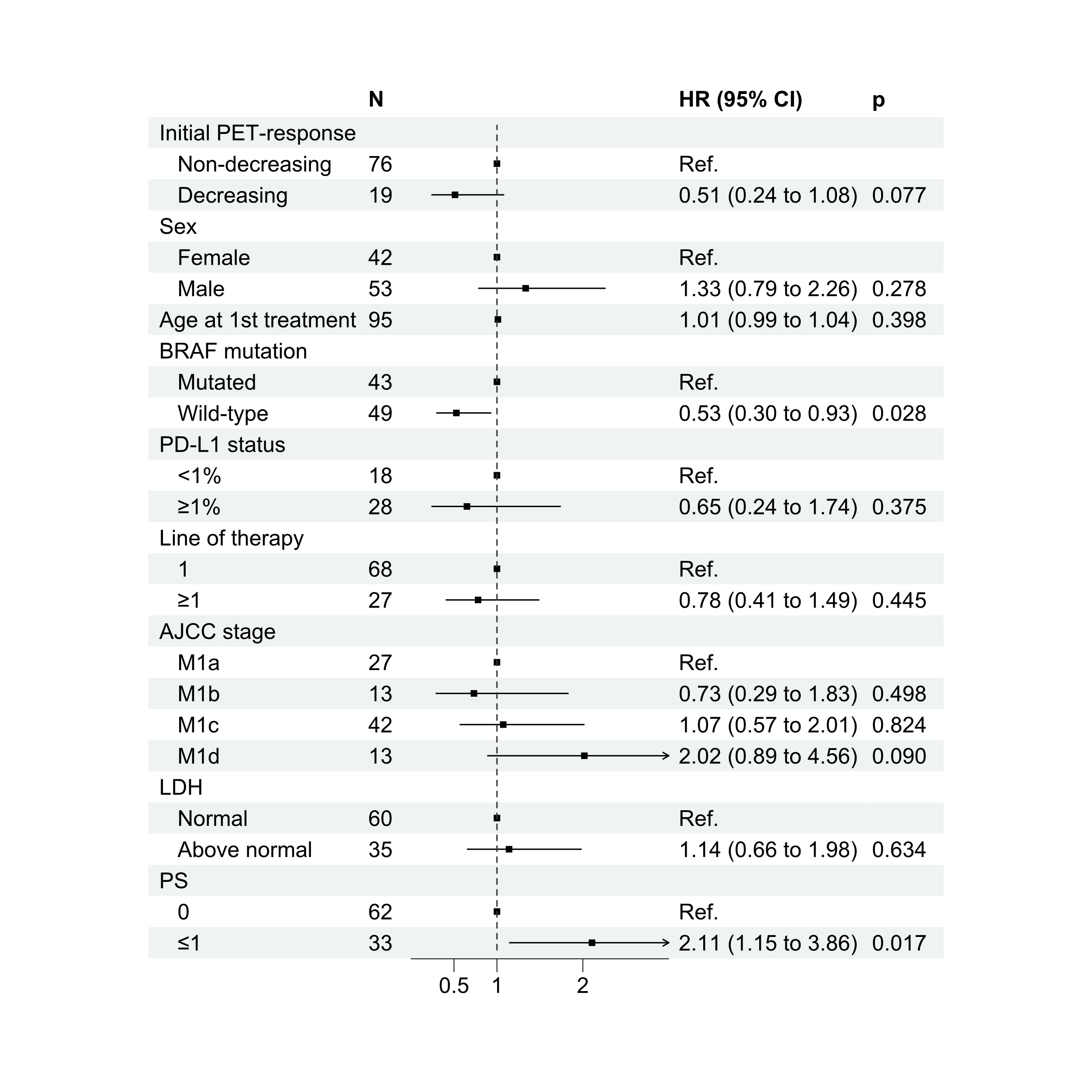


**Figure S3:** Subgroup multivariable Cox Regression analysis of PFS for patients with initial stable disease evaluated by PET-CT scan in the Eastern part of Denmark.

HR: hazard ratio; 95% CI: 95% confidence interval; PET-CT, Positron Emission Tomography Computerised Tomography; PD-L1, Programmed Cell Death Ligand 1; AJCC stage, American Joint Committee on Cancer stage; LDH, lactate dehydrogenase; PS, Eastern Cooperative Oncology Group performance status.
